# COVID-19 Vaccine Hesitancy and Implications for Economic Recovery: Evidence from Nelson Mandela Bay Municipality in South Africa

**DOI:** 10.3390/vaccines11081339

**Published:** 2023-08-07

**Authors:** Syden Mishi, Godfred Anakpo, Weliswa Matekenya, Nomonde Tshabalala

**Affiliations:** Department of Economics, Nelson Mandela University, Gqeberha 6001, South Africa; syden.mishi@mandela.ac.za (S.M.); weliswa.matekenya@mandela.ac.za (W.M.); nomonde.tshabalala@mandela.ac.za (N.T.)

**Keywords:** COVID-19, vaccine hesitancy, implication, consequences, economic recovery

## Abstract

The phenomenon of vaccine hesitancy is a growing threat to public health with far-reaching implications. The widening gap between the vaccinated and the proportion of vaccinated people needed for herd immunity raises two critical research questions that are of interest to practitioners, researchers, and policymakers: (1) What determines one’s decision to be vaccinated? (2) What is the implication of COVID-19 vaccine hesitancy for economic recovery? In this study, we use empirical data in the context of South Africa to investigate factors affecting COVID-19 vaccine hesitancy and their implications for economic recovery. Findings reveal key socio-demographic and institutional drivers of COVID-19 vaccine hesitancy, which include age (the youth are more hesitant), inadequate information on the vaccine (those who perceive they have adequate information are vaccinated), trust issues in government institutions, conspiracy beliefs, vaccine-related factors, and perceived side effects associated with the vaccine. Additionally, an individual’s decision to remain hesitant about COVID-19 vaccination has implications for businesses and the economy by limiting movement and trade, increasing unemployment, and causing a resurgence of new variants. Based on the findings, action plans such as information dissemination, convenience vaccination centers, consistent communications, and targeted campaign strategies are recommended for improving vaccine uptake and a positive economic recovery.

## 1. Introduction

Vaccine hesitancy is an increasing challenge to public health with far-reaching implications. The concept of vaccine hesitancy is used to refer to delay, indecisiveness, or refusal to take vaccines (despite their availability) to prevent or combat disease or infection [1,2,3,4]. Increasing vaccine uptake is an important pathway for achieving herd immunity for public safety [5]. According to Chevallier et al. [6], the proportion of the population that needs to take vaccines in order to reach herd immunity ranges from 75 to 90% (depending on factors such as the basic reproduction number, vaccine-induced immunity duration, and whether vaccines prevent transmission). Although vaccine hesitancy has existed for centuries [7], its negative effects have become more pronounced during the COVID-19 pandemic [8], and it is regarded as one of the most serious threats to global health and the economy at large [9]. This raises two important thought-provoking questions: first, what determines one’s decision to be vaccinated; second, what is the implication for economic recovery if herd immunity is not achieved due to a high level of COVID-19 vaccine hesitancy?

Past and present studies identified key determinants of vaccine hesitancy and underscored that vaccine hesitancy is a complex and often context-specific phenomenon that varies across time and place, and vaccines can be influenced by factors such as complacency, convenience, needle phobia, or a lack of understanding about how vaccines work [10,11]. Additionally, structural factors such as health inequalities, socio-economic disadvantages, systemic racism, and barriers to access are major contributors to vaccine skepticism and low uptake [4,8,12,13]. Dubé and MacDonald [14] documented that non-vaccine-related factors that influence an individual’s decision to be vaccinated may vary from one location to another and that contextualization is an important precursor and feedback for tailored vaccination programs.

Furthermore, vaccination hesitancy has implications for economic recovery. Returning to some normalcy and the recovery of economies is dependent on the success of measures such as vaccination. Even though vaccine coverage is steadily increasing across South Africa, the virus remains a threat. Vaccination uptake has implications for business, so the economy can fully open without repercussions, allowing people to freely move and conduct their daily activities, which is critical for business recovery. There is a dearth of literature focusing on the implications of vaccine hesitancy for economic recovery, especially in South Africa, where vaccine hesitancy is relatively high (at 41 percent of the adult population, which is above the global level of 33 percent) with dire impacts on the economy. The purpose of this study is, therefore, to identify the factors affecting COVID-19 vaccine hesitancy and their implications for economic recovery.

Following the introduction above, the rest of this paper is structured as follows: Section 2 documents a review of the available and related literature, such as vaccine hesitations, their determinants, and potential implications. Section 3 presents the methods used to describe the population, data collection procedure, and analysis. Section 4 and Section 5 report the results and discussions, respectively. The last section draws conclusions and recommendations based on the findings and highlights the limitations of the study.

## 2. Literature Review

The empirical studies in the COVID-19 hesitancy literature cover different locations/countries and are often associated with different socio-cultural backgrounds and characteristics. Vaccine hesitancy was higher among African Americans (US), accounting for approximately 34 percent, according to [15]. In most US states, Black and Hispanic Americans are receiving COVID-19 vaccinations at lower rates than White Americans [16]. These trends, in part, may be explained by a history of racial barriers and injustices in the American medical establishment. For example, people of colour report more frequent negative experiences with healthcare providers and lower rates of health insurance coverage than White Americans [16].

Vaccine hesitancy is associated with a host of factors, including socio-demographic factors such as sex, education, employment, income, having children at home, and the perceived risk of contracting COVID-19 [1,2]. Some of the literature has highlighted that females are less likely to be vaccinated, which contributes to vaccination hesitancy [1,17,18,19]. Other studies with similar findings include those by [2,20], who documented that women have greater vaccine hesitancy than their male counterparts. Women often bear most of the responsibility for family healthcare, so it may be that women are generally more aware of and concerned about the negative side effects of vaccinations and may therefore shy away from vaccination [18]. In addition, Black/Hispanic, lower-income, and larger households are less likely to be vaccinated [21]. The youth [20,22,23] and those with low monthly incomes are predicted to have vaccine hesitancy regardless of education level. There are mixed results for age on COVID-19 vaccine hesitancy, with studies showing both greater hesitancy for young people in some studies and no effect of age in others [24,25]. As people with low socio-economic status have been disproportionately impacted, this may explain, in part, the link the authors discovered between vaccine hesitancy, distrust, low monthly income, and ethnic disparity [20]. Robinson et al. [9] highlighted that lower income or education levels and belonging to an ethnic minority group were consistently associated with being less likely to intend to vaccinate. Low levels of educational attainment have been generally associated with greater vaccine hesitancy [26]. More educated people have greater skepticism of the scientific mechanisms of the medicine and its efficacy and safety, while less educated people may decline vaccination due to a lack of information.

Influences have been reported to emerge from a lack of knowledge, personal perception of the vaccine, or due to influences from one’s social/peer environment. In that context, Ref. [27] reported that low knowledge about COVID-19 also increases vaccination hesitancy. On a related issue, Ref. [28] found that conspiracy beliefs negatively predicted general attitudes toward vaccines. Knowledge and conspiracy beliefs may also influence one’s perception of the vaccines and, therefore, their decision to be vaccinated. This argument is supported by [29], who reported that individuals’ perceptions and attitudes play a significant role in the decision to vaccinate against COVID-19. Thus, individual barriers to vaccination include a lack of knowledge, perceptions, attitudes, and beliefs about science, vaccines, the health system, and the government. According to refs. [29,30], these perceptions are shaped by (mis)information exposure amplified by social media, the community, and the health system. Others delayed getting vaccinated because they wanted to see how vaccines worked on other people and pointed out potential side effects [2]. As a result of the preceding statement, vaccines are regarded as dangerous and lethal [29]. The authors further revealed that to reduce the detrimental consequences of conspiracy beliefs, exposure to anti-conspiracy arguments, both before and after exposure to conspiracy theories, can restore the vaccination intention [31,32]. Other factors, such as lack of trust in the vaccines and side effects, affect an individual’s decision to be vaccinated [33,34].

On the other hand, there are influences arising due to historical, socio-cultural, environmental, health system/institutional, economic, or political factors. Amit et al. [29] also stated that political issues play a role in vaccination hesitancy. In their study on vaccine hesitancy in South Africa, ref. [2] stated that political factors will play a significant role in shaping attitudes toward COVID-19 vaccination. Political discontent or disillusionment may play a role; people who had positive attitudes toward the government in general and its handling of COVID-19 were more likely to accept COVID-19 vaccination. The structural barriers are vaccine procurement, supply, and logistics, as well as media and policy issues. Katoto et al. [20] discovered that among respondents, government distrust was associated with vaccine hesitancy. Political affiliation affected the perception of the risk of contracting COVID-19 and was also a significant predictor of vaccine hesitancy [20,23].

The findings of [35] suggest that vaccination attitudes are influenced not only by students’ level of health knowledge but also by other motivational and psychological factors, such as a sense of individual responsibility for population health and a common sense about the value of civic life and social solidarity, as demonstrated by other studies on the COVID-19 pandemic and previous emergencies. Ryan and Malinga [36] documented that vaccine-hesitant individuals are heterogeneous and vaccine hesitancy can arise in a variety of contexts, necessitating that interventions to address vaccine hesitancy be context- and issues-specific. Similarly, Wiysonge et al. [37] also found from their empirical evidence on vaccination hesitancy in South Africa that individuals’ vaccination attitudes and practices are often the result of a continuing engagement that is based on evolving personal and societal circumstances, which may alter over time.

The above literature provides evidence that non-vaccine-related factors that influence an individual’s decision to be vaccinated may vary from one location to another and that contextualization is an important precursor and feedback for tailored vaccination programs. Furthermore, the implications of vaccine hesitancy for economic recovery have not been well explored in the literature. Therefore, this study seeks to explore the determinants of vaccine hesitancy and its implications using the case of the Nelson Mandela Bay Municipality of South Africa.

## 3. Methods

The empirical data for this analysis was drawn from the Nelson Mandela Bay Municipality. The target population was all adults in the Nelson Mandela Bay Municipality, and the three major towns of Gqeberha, Kariega, and Despatch were considered. In 2019, the Nelson Mandela Bay Metropolitan Municipality was comprised of 375,000 households, and when using Raosoft’s sample calculation (Raosoft is *a software company that primarily calculates or generates the sample size of a research or survey* (*Raosoft Inc.*, *Seattle*, *WA*, *USA*, *2004*)), a minimum of 400 respondents across Gqeberha, Kariega, and Despatch are considered representative. The survey was designed, administered, and interviews were held in these three regions of the Bay to ensure representation and to consider other demographics such as population group, age, and gender. The composition of households by population group consists of 62.9 percent, which is attributed to the African population group. The coloured population group had a total composition of 19.6 percent (ranking second), the White population group had a total composition of 16.2 percent of the total households, and the smallest population group by households was the Asian population group, with only 1.3 percent in 2019.

The interview questions were prepared by the authors based on the objectives of the study and the relevant literature. It covered a set of questions on four main areas: (1) socio-economic characteristics; (2) sources of information and contextual influence; (3) individual and group influence; and (4) questions related to the vaccine or vaccination. A pilot test was conducted with some prospective participants in the study area (on 1 and 2 March 2022) to identify any weaknesses with the instrument, thereby strengthening its validity. Given that the three major towns of the municipality were represented and homogenous in each area, a convenience sampling technique was employed, and those who were available and willing to respond to the questionnaire participated in the interviews and online survey from 4–30 March 2022. A convenience sample is a type of non-probability sampling method where the sample is taken from a target population that meets selection criteria or certain research criteria, such as being easily accessible, geographically convenient, available at a given time, willing to participate in the interview or survey, and being included for the purpose of the study [38,39]. A total of 460 respondents were selected for this study in the above thematic areas. Since the study is qualitative in nature, descriptive statistics, univariate analysis, graphical presentation, and thematic categorization were used for the analysis.

The second part on the implications of the economic recovery, given the updated/new preferences among individuals and groups of people, was approached in two steps. The first was to establish the economic (business, job, and cost) and public health consequences of COVID-19 vaccine hesitancy. This was achieved through a deduction process from the incidence of vaccine hesitancy and how hesitancy influences the behavior of individuals.

## 4. Results

Descriptive statistics were used to describe the socio-demographic and other variables of interest, such as COVID-19 and other vaccine hesitancy questions. Results on socio-demographic characteristics and general information regarding accepting vaccines when recommended by a health care worker, beliefs on whether there are any better solutions or treatments other than the COVID-19 vaccine, their perception of the safety of the COVID-19 vaccine, whether the government should make COVID-19 compulsory, their trust in the government, the role of health care workers, their experience with the COVID-19 vaccine, and informational needs about the vaccine were reported. Table 1 documents summary statistics on the background characteristics of the participants.

The table shows that out of the total sample population that was involved in the study, about 29 percent of the study participants were male, and 71 percent were female. This may be partly due to the fact that females constitute slightly over half the total population in the municipality and mainly due to the availability of more females than their male counterparts for the interview. The majority of the respondents were in the age group between 25 and 34 years (37%), followed by 18–24 years (31%), 35–54 years (26%), and the lower population groups of 55–64 years and 65+ years (5% and 1%, respectively). Concerning education, most of the respondents have tertiary education and matriculation, representing 46 percent and 43 percent, respectively, while 10 percent responded to having primary education, and a small number (1%) had no education. The majority, 75 percent of the study, reported that they were single, with 17 percent being married and a few being divorced, widowed, and/or cohabiting. Most of the respondents reported that they belong to the Christian religion (79%), and very few belong to other religious groups. In terms of representation of ethnic groups, about 87 percent of the participants reported that they were Africans; a few were coloured, Asian or Indian, and White. The table also shows that most of the respondents are based in Gqeberha (59%), followed by Kariega (18%), and a few are from Despatch and other places. The socio-economic status of the respondents was primarily from households whose incomes were average. The average income in this context means the same as the income of most households; less than average means less than the income of most households; and above means over the income of most households. Therefore, the results show that 59% of households have an average income, followed by 38 percent whose income is lower than the average, and very few have an above-average household income.

Results on vaccination information, knowledge, and attitude related to the vaccine are reported in Table 2.

When asked if they are vaccinated, most of the participants (48%) responded that they are not vaccinated and that they are not planning to get vaccinated, while 30 percent are still not vaccinated but are planning to get vaccinated but are delaying it. This shows the high level of vaccine hesitancy (79%) among the respondents. Only 20 percent reported that they were fully vaccinated, and 2 percent reported receiving only one vaccination. Regarding the subjective care characteristics, respondents show that 69 percent responded that they are as careful and responsible as others, while 28 percent responded that they are extra-careful, and very few people said they are not careful.

The previous sections confirmed the existence of COVID-19 vaccine hesitance within Nelson Mandela Bay. Of interest, then, is what the possible drivers of such behaviors are. The authors started by inquiring whether one had ever refused any other vaccination (other than the COVID-19 vaccine). A surprising majority (58% of the sample) had not refused any other vaccines in the past, yet, as depicted, the majority are not willing to be vaccinated against COVID-19.

Table 2 also shows that 94 percent of the participants have heard about the COVID-19 vaccine, while only 6 percent responded “No”, which means that they have not heard about the vaccine. In one question, where respondents were asked whether the government should make vaccines compulsory, bearing in mind that not all those who disagree are unvaccinated, most of the participants, representing 68 percent, were against it, while the participants that were in favour were 10 percent; the others who responded preferred not to respond, and those who did not know were 14 percent and 8 percent, respectively. This means that more people are against vaccine enforcement (compulsory vaccination), a sign of high hesitance toward the vaccine! The variable was recorded for further analysis, including the vaccine hesitance ratio.

The beliefs or perceptions around the safety of the vaccine show that 47 percent of respondents do not believe that vaccines are safe for them, 35 percent prefer not to respond, and 18 percent believe vaccines are safe for them. This shows that there is a greater sense of not trusting the safety of the vaccine among the respondents.

The majority of respondents do not believe that vaccine is the best solution for COVID-19; this is shown by 24 percent who believe there is another solution than vaccination, while 60 percent of the sample who responded are not sure, and only 16 percent (less than 1 in every 5) believe that vaccine is the best solution for COVID-19. This shows high hesitancy on COVID-19 if the not-sure and yes respondents are combined.

Concerning reasons for refusing any vaccination in the past, some cited that it was not needed (10%), there was a lack of sufficient information on the vaccine (13%), the vaccine was not effective (10%), the vaccine was not safe (12%), and the possible side effects (15%) as the reasons for being unwilling to be vaccinated (Table 3).

Different strategies have been used to decrease COVID-19 vaccine hesitancy, including information packages disseminated via various platforms and role models (popular personalities), among others. The results to the question Do you find the information conveyed by popular personalities (local and international) helpful? are shown in Table 4, which shows that 35 percent of the information conveyed by popular personalities is somewhat helpful, with 16 percent believing that they are very helpful and 26 percent not sure.

Management of the pandemic needs to win the trust of the population for buy-in on its effects to address it. Only 19 percent believe that the side effects of the vaccine are being appropriately tracked, with 34 percent responding no. The majority of respondents, 48 percent, highlighted that they do not trust the government. A third of the sample considers that, in general, individuals follow a wait-and-see approach; this is common when available information is inadequate and/or not trusted. This is like experimenting with fellow members of the community, a kind of approach where “if nothing happens to others, then I will follow suit”. Approximately 31 percent believe that those vaccinated are mostly vaccinated due to social pressure.

Based on the data sample, almost 97 percent of respondents have heard about the COVID-19 vaccine, while only 3 percent claim to have no knowledge about the vaccine. Hearing about the vaccine is dependent on how such information is disseminated and the strategies used to implement the vaccine rollout. The internet and social media platforms have been leveraged to distribute COVID-19 vaccine information nationwide.

About 19% of the participants believe that the actions of one person can affect others, while about 22 percent fear that someone closer to them, such as workmates, schoolmates, or family, can infect them with COVID-19. At the peak of COVID-19, many people were interested in attending gatherings; it may well have been justified by the thought that someone they knew was unlikely to infect me. About 26 percent are traveling less for fear of being exposed by those not vaccinated, while 26% feel reluctant to go out shopping due to fear of being infected (Table 5).

The results (Table 6) of the logistic model show that generally, the youth age group believes that there is a better alternative treatment for COVID-19 compared with older people, which means there is higher hesitancy amongst the youth group. Females believe that there is no better alternative treatment for COVID-19 compared with males; therefore, there is less hesitancy among females compared with males. The religious factor shows that Christians are more likely to believe that there are better alternative treatments for COVID-19 compared with other religious groups, which means that Christians are more hesitant to take the vaccine. Higher-educated respondents believe that there is no better alternative treatment compared with non-tertiary respondents, which shows that among tertiary respondents, there is less hesitancy to take the vaccine. High-income earners believe that there is a better alternative treatment compared with low-income earners, which shows high hesitancy among the high-income group.

The results (Table 7) of the study show that generally, youth find, or believe, that the COVID-19 vaccine is not safe compared to older people, which means that they are more hesitant. Females are more likely to believe that vaccines are safe compared to males, and this means that females are less hesitant than males. The religion factor shows that Christians are more likely to find or believe the vaccine is not safe compared with other religious groups, and this means that they are more hesitant. Respondents who are at the tertiary level are more likely to believe the vaccine is not safe compared with respondents who are non-tertiary, and this means that educated people are more hesitant. High-income earners are more likely to believe that vaccines are not safe for them compared to lower-income earners, which means that high-income groups are likely to be hesitant to be vaccinated.

## 5. Discussions

Socio-demographic factors such as sex, education, employment, income, having children at home, and the perceived risk of contracting COVID-19 were all significant predictors of vaccine hesitancy. Findings show that females are more hesitant to receive vaccination than males, which is consistent with some empirical conclusions in the extant literature. Some of the literature has highlighted that females are less likely to be vaccinated, which contributes to vaccination hesitancy [1,17,18,19]. Similarly, women may be more hesitant than men to receive COVID-19 vaccines [2,20] though they are more susceptible to the virus [40]. Ref. [18] reported that women have greater vaccine hesitancy than men. Women often bear most of the responsibility for family healthcare, so it may be that women are generally more aware of and concerned about the negative side effects of vaccinations, which may keep them from performing essential tasks due to illness [18].

In addition, there are mixed results for age on COVID-19 vaccine hesitancy, with studies showing both greater hesitancy for young people in some studies and no effect of age in others [24,25]. As people with low socio-economic status have been disproportionately impacted, this may explain, in part, the link between vaccine hesitancy, distrust, low monthly income, and ethnic disparity [20]. Low levels of educational attainment have been generally associated with greater vaccine hesitancy [26]. Additionally, more educated people have greater skepticism of the scientific mechanisms of medicine and its efficacy and safety, while less educated people may decline vaccination due to a lack of information.

Furthermore, factors such as lack of knowledge, personal perception of the vaccine, or influences of the social or peer environment are some predictors of vaccination hesitancy. Other studies, such as [27], reported that insufficient knowledge about COVID-19 could lead to vaccination hesitancy. On a related matter, ref. [28] documented that conspiracy beliefs associated with the vaccine may predict negative attitudes toward vaccines. Similarly, ref. [29] highlighted that individuals’ perceptions and attitudes play a significant role in one’s vaccination decision. The authors also documented that individual barriers to vaccination include a lack of knowledge, perceptions, attitudes, and beliefs about science, vaccines, the health system, and the government. According to Amit et al. [29], these perceptions are shaped by misinformation exposure amplified by social media, the community, and the health system. Depending on how one feels about vaccines, one’s social network may have a positive or negative impact on vaccination uptake. As a result, interpersonal barriers, such as networks and social capital, influence health beliefs and decisions. Other studies have found that negative interactions with the healthcare system may lead to vaccine hesitancy [20,41]. Others delayed getting vaccinated because they wanted to see how vaccines worked on other people and pointed out potential side effects [2,42]. As a result of the preceding statement, vaccines are regarded as dangerous and lethal [29]. The authors further revealed that to reduce the detrimental consequences of conspiracy beliefs, exposure to anti-conspiracy arguments, both before and after exposure to conspiracy theories, can restore the vaccination intention [31,32].

Participants believed that this vaccine used the same virus to ‘immunize’ an individual’s system, which could have unintended consequences. Other participants cited that this specific brand was not recognized by other countries, and therefore they wanted and waited for other vaccines [29]. Meanwhile, others refused to receive vaccines due to beliefs about their safety and effectiveness [29]. Another reason for COVID-19 vaccination hesitancy is a lack of trust due to the belief that the vaccine moved too quickly through clinical trials, and there is a widespread belief that vaccination is extremely risky, resulting in serious health consequences [1,11]. Similarly, according to Khubchandani et al. [15], participants in America believed that socio-political factors and pressures could lead to a rushed approval of the COVID-19 vaccine without assurances of safety and efficacy. Furthermore, refs. [33,34] found, in their separate studies, that the psychological factors that affect COVID-19 vaccination hesitancy include trust in the vaccine and fear of side effects.

The role of health care workers in providing the relevant information regarding the COVID-19 vaccine increases the likelihood of respondents believing that the vaccine is safe compared with those who were not satisfied with the information provided by health care workers; this means that they are likely to be less hesitant. The respondents have highlighted that those who trust the government are likely to believe that the vaccine is not safe for them compared with those who do not trust the government. Those that are pressured by their social network believe that the vaccine is not safe compared with the respondents that are not pressured by social networks. Those who know which vaccine to choose from are more likely not to believe that the vaccine is safe compared to those who do not know which vaccine to choose. On the other hand, there are influences arising due to historical, socio-cultural, environmental, health system/institutional, economic, or political factors. The finding supports similar findings by [29] that politics may significantly affect vaccination hesitancy. In their study on vaccine hesitancy in South Africa, ref. [2] stated that political factors will play a significant role in shaping attitudes toward COVID-19 vaccination. Political discontent or disillusionment may cause people to have ill feelings and cast doubt on the government in handling information related to the vaccination and the vaccine itself. The structural barriers are vaccine procurement, supply, and logistics, as well as media and policy issues. A similar conclusion was made by [20], who reported that distrust in government is associated with vaccine hesitancy.

According to [35], factors beyond health knowledge, including psychological factors, such as a sense one responsibility for others’ health and a common sense of solidarity and belongingness, play a great role in vaccination hesitancy. Not only the general public but also those pursuing a career as a health professional appear to be struggling to keep up with a growing body of evidence, increasingly complex information, and conflicting feelings about vaccines. Therefore, public health information campaigns should also be supported by other actions aimed at raising students’ consciousness regarding the crucial role of individuals’ engagement in safeguarding their own and others’ health through vaccination. Other reasons for COVID-19 vaccination hesitancy include that the time frame between vaccine development, production, and availability was too short, the politics surrounding the vaccine development process, misinformation from social media, previous COVID-19 infection or health conditions, health experts, and pharmaceutical companies [1,20,43]. Concerning that [37] also documented from their empirical evidence on vaccination hesitancy in South Africa, one’s attitude towards vaccination is the issue of active engagement that is based on evolving personal and societal circumstances, which may alter over time. The authors added that as COVID-19 vaccination expands internationally, scientists and policymakers must examine the extent and drivers of vaccine hesitancy in each environment to design specialized and focused methods to combat it.

## 6. Implications for Economic Recovery

The behavior of individuals has implications for the rest of the economy, as is the case with vaccination. Due to the inherent risk of the pandemic and the fact that an individual cannot easily identify where the risk lies, risk aversion becomes the dominant strategy. A risk-averse individual does their best to avoid a risky environment, decision, or situation, while a risk-taker has no restraint. Given the much-publicized effects of COVID-19 in terms of sickness, hospitalization, and death, any option to avoid infection is likely to be appreciated by risk-averse individuals. Therefore, the vaccinated are likely to be risk averse; they prefer the safer option of the benefit that comes with vaccination (no matter how small it is) to experiencing the worst. Although one could argue that taking the vaccine is the riskier decision given the myths and misinformation, the limited cases of hospitalization and death of the vaccinated are enough to warrant vaccination as a safer decision for a risk-averse individual.

Taking that the unvaccinated are risk averse, it is sufficient to say they have limited restraint in traveling and going out as per the prior COVID-19 period; on the other hand, the vaccinated, the more risk averse, are likely to be fearful of going out, given the knowledge that there are some who are unvaccinated. By intuition, one gets vaccinated because they believe the pandemic is real, not fiction; therefore, the risk of going out is higher. When movement is restrained, it affects businesses that rely on the movement of people, such as sit-in restaurants, the tourism sector, the transport sector, and many others (including the informal sector).

Furthermore, vaccination hesitation may negatively affect economic recovery through international trade constraints. The outbreak of the COVID-19 pandemic has defined and shaped international and travel laws, rules, and policy in two phases; the first was the complete closure of borders following the outbreak of the pandemic, thus disrupting business activities, livelihoods, international trade [44,45,46,47,48], and productivity [45,49,50] across various sectors [51]. The second phase was the opening of international borders (for instance, airports) to those who had taken the COVID-19 vaccine following its development and availability. Thus, vaccine hesitancy will compromise international trade, travel, and business activities, posing a potential threat to economic recovery, especially in low- and middle-income countries.

Additionally, economic recovery may also be affected by the unemployment of the unvaccinated. As part of preventive measures, some companies have made vaccination a mandatory exercise for all employees. Consequently, employees who fail to take the vaccine are given automatic exit from the company, which may lead to unemployment, low productivity, and a low economic recovery. Vaccination hesitancy in low- and middle-income countries is more likely to foster the development of new variants that could overcome current vaccines, which may cause another economic downturn.

If vaccine hesitancy, which this study confirms exists, negatively affects businesses, this means recovery is going to take longer than projected, with repercussions on household income and overall wellbeing, especially in low-income countries where poverty and inequality are already deep and wide [52,53]. Strategies need to be developed on how to improve vaccine uptake, and as argued above, it starts with understanding the social structure and the trusted sources of information; that trust is the greatest asset of many individuals and communities, and they rely on it to make decisions. This is backed by social network theory, where the information held by the most central person (the nucleus of the network) easily diffuses and is more positively received than information shared by strangers or any less trusted sources (peripheral elements).

As mentioned in the background, many governments and authorities have made an effort to study the behavior of individuals by setting up behavioral insight units. These track the mood of the populace and how individuals are likely to respond to policy changes and various calls to action. That helps with a higher uptake, support, and effectiveness of policies or interventions. The Western Cape Provincial Government had established such a unit, which spearheaded behavioral change when the water crisis day zero was imminent; their efforts were successful. Many programs are ongoing and guided by such behavioral insights. The business chamber can play a role in establishing one for the benefit of the business community and the general community. The insights are required to guide recovery efforts.

## 7. Conclusions

Hesitancy toward COVID-19 vaccination is a major threat to public health with far-reaching implications. This study uses empirical data in the context of South Africa to examine factors affecting COVID-19 vaccine hesitancy and their implications for economic recovery. Findings reveal key socio-demographic and institutional drivers of COVD-9 vaccine hesitancy, which include age (the younger are more hesitant than the older generation), inadequate information received about the vaccine (those who perceive they have adequate information are vaccinated), trust issues in government institutions, conspiracy beliefs, vaccine-related factors, and perceived side effects associated with the vaccine. Additionally, an individual’s decision to remain hesitant about COVID-19 vaccination has implications for businesses and the economy by limiting movement and trade, increasing unemployment, and causing a resurgence of new variants. The decision to vaccinate is mainly affected by family members rather than information received on other platforms or from any other person. This also has implications for the intervention strategies that build trust in the public, such as sufficient and effective communication with credible institutions that provide consistent information regarding the vaccine, its side effects, and conspiracy beliefs surrounding it. These strategies should effectively address community concerns with side effects and potential health risks by disseminating information and increasing the population’s knowledge of the vaccine, in addition to its risk factors, effectiveness, and side effects. Other reliable institutions for vaccine information include health workers and mass media (TV and Radio). Other action plans, such as information dissemination, convenience vaccination centers, consistent communications, and targeted campaign strategies, are recommended for improving vaccine uptake and a positive economic recovery.

Based on the findings, future research that focuses on areas such as the role of information, information sources, and institutions in vaccine hesitancy is suggested.

## 8. Limitation

There are some limitations to this study. First, the participants were selected from the Nelson Mandela Bay Municipality of South Africa, which is not fully representative of the entire population of the country, thus limiting the generalization of the findings to the whole population. However, lessons from this study can serve as a reference point and are, to some extent, applicable in other contexts. Second, the qualitative nature of the data does not allow a more rigorous statistical analysis; however, the study utilizes the in-depth information from the interview as leverage to address the study objective with accuracy.

## Figures and Tables

**Table 1:** Demographic characteristics.

Variables/Categories	Percentages (%)
**Gender**	
Male	29
Female	71
**Population group**	
African	87
Coloured	9
Asian/Indian	1
White	2
Other race	1
**Age**	
18–24	31
25–34	37
35–54	26
55–64	5
65+	1
**Education**	
No formal education	1
Primary	10
Matric	43
Tertiary	46
**Religion**	
Christian	79
Muslim	3
Traditional	13
Others	5
**Marital status**	
Single	75
Married	17
Divorced	3
Widowed	3
Cohabiting	2
Other	0
**Township**	
Gqeberha	59
Kariega (Uitenhage)	18
Dispatch	8
Other places	15
**Dwelling Type**	
Free standing	15
Townhouse/complex/flat	24
Multiple shared house	55
Others	6
**Income**	
Lower than average	38
On average	59
Higher than average	3

**Table 2:** Vaccination information, knowledge, and attitude related to the vaccine.

	Percentages (%)
**Vaccination information**	
Fully vaccinated	20
Partly vaccinated (Yet to go for 2nd shot)	2
Not vaccinated but will do so in the future	30
No intention to be vaccinated at al	48
**Awareness about COVID-19 vaccine**	
Yes	94
No	6
**One’s perception of vaccine safety**	
It is safe	18
Not safe	47
Prefer not to say it	35
**Views on compulsory vaccination**	
Yes	10
No	68
Don’t know	8
Prefer not to say it	14
**Ever refused vaccination in the past?**	
Yes	42
No	58
**Perceived availability of alternative COVID-19 vaccines**	
Yes	16
No	24
Not sure	60
**Subjective care**	
Am extra careful	28
As good as others	69
Not as careful as others	3

**Table 3:** Reasons for refusing any vaccination in the past.

	Freq.	Percentages
I never refused a vaccine recommended by a healthcare worker	198	33
Did not think it was needed	61	10
Did not have enough information on the vaccine	80	13
Did not think the vaccine was effective	58	10
Did not think the vaccine was safe	74	12
I was concerned about side effects	93	15
I had a bad experience with a previous vaccination	23	4
Did not know where to get the vaccination	6	1
Other logistic problems	15	2

**Table 4:** Social trust and vaccine hesitancy.

Variables	Percentages (%)
**Are side effect being tracked?**	
Yes	19
No	34
Not sure	39
Prefer not to say it	8
**Information by key personalities helpful?**	
Somewhat helpful	35
Very helpful	16
Not sure	26
Not helpful	23
**Preferring to wait to see others**	
Yes	29
No	22
Not sure	22
Prefer not to say it	27
**Trust government is making decision in my best interest**	
Yes	16
No	48
Prefer not to say it	36
**Social pressure to be vaccinated**	
Yes	31
No	36
Prefer not to say it	33

**Table 5:** Risk perception and decision on mobility.

Variables/Questions	Percentages (%)
**Own action affect others?**	
Yes	19
No	47
Prefer not to say it	34
**Fear that someone close may get infected**	
Yes	22
No	34
Prefer not to say it	44
**Travelling less in fear of unvaccinated**	
Yes	26
No	50
Prefer not to say it	24
**Going out less for shopping in fear**	
Yes	21
No	53
Prefer not to say it	26

**Table 6:** Determinants of the probability of hesitancy (hesitancy based on safety concerns). Multivariate analysis.

Hesitancy Safety	Coef.	St. Err.	*t*-Value	*p*-Value	[95% Conf	Interval]	Sig
Age	−0.065	0.271	−0.24	0.811	−0.596	0.467	
Gender	0.49	0.289	1.69	0.09	−0.077	1.057	*
Religion: Christian	0.073	0.332	0.22	0.826	−0.578	0.725	
Education	−0.32	0.31	−1.03	0.302	−0.929	0.288	
Income	0.015	0.29	0.05	0.959	−0.553	0.583	
Infor	−0.063	0.302	−0.21	0.835	−0.655	0.529	
Satisfied	1.642	0.327	5.03	0	1.002	2.282	***
Government	−0.357	0.309	−1.15	0.248	−0.964	0.249	
Social	−0.056	0.274	−0.20	0.839	−0.592	0.481	
Choice	−0.133	0.295	−0.45	0.653	−0.71	0.445	
Constant	−2.376	0.305	−7.78	0	−2.974	−1.778	***
Mean dependent var	0.178		SD dependent var			0.383	
Pseudo r-squared	0.102		Number of obs			460	
Chi-square	44.176		Prob > chi2			0.000	
Akaike crit. (AIC)	409.070		Bayesian crit. (BIC)			454.514	

*** *p* < 0.01, * *p* < 0.1.

**Table 7:** Factors explaining vaccine hesitance (hesitancy based on belief of alternative ways). Multivariate analysis.

Hesitancy Alternative	Coef.	St. Err.	*t*-Value	*p*-Value	[95% Conf	Interval]	Sig
Age	−0.432	0.298	−1.45	0.148	−1.017	0.153	
Gender	0.686	0.297	2.31	0.021	0.104	1.268	**
Religion: Christian	−0.131	0.346	−0.38	0.706	−0.809	0.548	
Education	0.126	0.318	0.40	0.691	−0.496	0.748	
Income	−0.37	0.304	−1.22	0.223	−0.965	0.225	
Infor	0.869	0.299	2.90	0.004	0.282	1.455	***
Satisfied	1.356	0.317	4.27	0	0.734	1.977	***
Government	1.418	0.298	4.76	0	0.835	2.001	***
Social	−0.117	0.295	−0.39	0.693	−0.695	0.462	
Choice	1.581	0.29	5.46	0	1.013	2.149	***
Constant	−3.085	0.369	−8.37	0	−3.808	−2.363	***
Mean dependent var	0.261		SD dependent var			0.440	
Pseudo r-squared	0.366		Number of obs			460	
Chi-square	193.310		Prob > chi2			0.000	
Akaike crit. (AIC)	356.737		Bayesian crit. (BIC)			402.181	

*** *p* < 0.01, ** *p* < 0.05.

## Data Availability

Data sharing is not applicable to this article due to the qualitative nature of the collected data.
